# FDG-PET staging and importance of lymph node SUV in head and neck cancer

**DOI:** 10.1186/1758-3284-2-19

**Published:** 2010-07-16

**Authors:** Gregory J Kubicek, Collin Champ, Shannon Fogh, Fen Wang, Eashwer Reddy, Charles Intenzo, Reginald W Dusing, Mitchell Machtay

**Affiliations:** 1 Thomas Jefferson University Hospital, Philadelphia, PA, USA; 2 Kansas University Medical Center, Kansas City, KS, USA; 3 University of California, San Fransisco, CA, USA; 4 University of San Francisco, San Francisco, CA, USA; 5 Case Western University Hospital, Cleveland, OH, USA

## Abstract

**Objectives:**

The role of positron emission tomography (PET) with fluoro-deoxy-glucose (FDG) in the staging of head and neck cancer (HNC) is unclear. The NCCN guidelines do not recommend FDG-PET as a part of standard workup. The purpose of this report is to examine the role of FDG-PET imaging in altering management and providing prognostic information for HNC.

**Methods:**

Retrospective review of HNC patients who had a staging FDG-PET scan performed at either Thomas Jefferson University or University of Kansas Medical Center between the years 2001 and 2007. A total of 212 PET scans were performed in patients who went on to receive radiotherapy.

**Results:**

The median follow-up time for all patients was 469 days. The PPV and NPV of PET imaging to correctly identify lymph node status was 94% and 89% respectively. Lymph nodes with extracapsular extension (ECE) had higher SUVs than nodes without ECE, 11.0 vs. 5.0 (p < 0.0007). Maximum SUV for the primary tumor > 8.0 was predictive of worse overall survival (p < 0.045), while the SUV of the lymph nodes was predictive for distant recurrence at one year--with a mean SUV value of 10.4 for patients with distant failure vs. 7.0 without (p < 0.05).

**Conclusions:**

FDG-PET staging in head and neck cancer has good positive and negative predictive values in determining lymph node status. The maximum SUV of the primary tumor is predictive of overall survival. This is the first report to find that the SUV of a lymph node is predictive for ECE and also for distant recurrence.

## Introduction

Approximately 47,500 new cases of head and neck cancer (HNC) are diagnosed annually in the United States [1], the cure rates for HNC, especially in advanced disease, remains poor [2]. Because of this there have been efforts to improve outcomes through the use of targeted agents [3], new radiation techniques such as intensity modulated radiation therapy (IMRT) and image guided radiation therapy (IGRT), as well as new types of diagnostic imaging. Scanning utilizing FDG has become standard of care for many malignant neoplasms [4] for its ability to more accurately stage tumors, and thus alter therapeutic decision making, compared to conventional imaging modalities such as computed tomography (CT) and magnetic resonance imaging (MRI).

The use of FDG-PET scanning in head and neck cancer is more controversial. NCCN guidelines do not include PET scans as part of routine staging, and many institutions use MRI or ultrasound guided biopsies rather than PET scans. The Radiation Therapy Oncology Group (RTOG) utilizes the clinical exam, chest x-ray, and pre-treatment CT scan as their standard workup.

Despite the lack of acceptance of staging PET scans into guidelines and cooperative groups, there are a number of studies that have examined the role of PET scans in HNC staging. Several studies [5-8] have looked at the value of PET in initial staging and the finding of metastatic disease or secondary cancers; the incidence seen in some of the larger studies [7,8] is 11.1 to 12.9%. The staging of lymph node status with PET has also been examined [5,9-17]; the sensitivity of PET in this regard ranges from 47 to 100% while the specificity ranges from 87 to 100%.

The prognostic value of SUVs is another unresolved issue with PET scanning. In theory, a greater SUV would correspond with a more aggressive tumor. While some authors [18-24] have found a relationship between maximum tumor SUV and clinical outcome, others [25,26] have not found this to be true. A few reports have examined the role of nodal SUV and outcome [18,19,27], with inconclusive results. Having prognostic information, especially in terms of patterns of failure, would be very useful. If there was a way to determine which patients had a greater risk of distant failure versus local failure, clinicians would be able to determine who would be ideal candidates for induction chemotherapy.

One of difficulties in interpreting the large number of studies on the topic of PET scans in the staging of HNC is the low number of patients in any one study. To increase the patient numbers we have combined the databases of two institutions with high-volume HNC services. This allows for better assessment of the capabilities of PET scans for staging HNC.

## Methods

This is a retrospective review of PET scans used in staging at two academic institutions; Thomas Jefferson University Hospital (Philadelphia, PA) and the Kansas University Medical Center (Kansas City, KS). Prior to beginning the review, approval was granted from the institutional review board (IRB) of each institution. Using the PET scan registry of both institutions, all of the PET scans for HNC that had been performed from 2000 to 2007 were analyzed. Patients who went on to have adjuvant or definitive radiotherapy with or without chemotherapy were included in the final analysis.

Images were acquired with either a PET or PET coupled with a low energy CT utilized for attenuation correction (CTAC) of the PET data. PET scans performed at Thomas Jefferson University Hospital used a Siemens Biograph 6 (Siemens Medical Solutions USA, Inc, Malvern, PA). For the PET/CTAC images, the CT component was a GE Lightspeed, 16 slice scanner (General Electric, Milwaukee, WI). Kansas University Medical Center used a Siemens ECAT ART (Advanced Rotational Tomograph) and a GE Discovery ST 16 slice scanner. Imaging protocols were similar at both institutions. Before the PET scan, all patients were required to fast for four hours, and blood glucose had to be less than 180 mg/dl. Ten to 15 mCurie (370-555 MBq) of (18F)-FDG was injected and 45 to 60 minutes was allowed for uptake before patient imaging. Patients were instructed to minimize any talking, chewing, swallowing, or movement of the head.

For fused PET/CT images, the CTAC and PET images were hardware coregistered in a single session. Non-contrast-enhanced CT imaging for attenuation correction and anatomic correlation was performed first from the vertex of the skull to below the kidneys. First the CT scan was completed using 180-mA tube current, 140-kV tube voltage, 0.5-second tube rotation, helical pitch of 1:1, and reconstructed slice thickness of 4.25 mm. The CT portion was acquired in less than 30 seconds. Immediately after the CT scan, a PET scan was acquired starting at the skull vertex with an acquisition time of 5 minutes per bed position with a one-slice overlap at the borders of the 14.6-cm field of view. Data were reconstructed using OSEM iterative reconstruction with two iterations and 28 subsets. Post-processing with a post filter at 5.45 mm full width at half maximum (FWHM), and a loop filter at 3.91 mm FWHM on a 128 × 128 matrix was then carried out. Images were viewed on a Xeleris (GE medical Systems) workstation. For scans that did not have CT fusion, the PET scan data was read in conjunction with other radiology exams (for example CT scan or MRI) if available.

Interpretation of the images consisted of reviewing the nuclear medicine report, information was gathered concerning areas of increased uptake and, if available, the maximum SUV. Radiology review was performed independently of reviewing the clinical chart. Clinical information was obtained from a retrospective chart review. Analysis was performed after all of the pertinent clinical and radiographic information had been gathered. Statistical analysis was performed using Graphpad (GraphPad Software Inc. La Jolla, CA) software.

## Results

A total of 212 patients (212 PET scans) were analyzed in this study, patient characteristics are outlined in Table 1. The majority of patients had advanced HNC, 80.2% were either stage III or IV. The median follow-up for all patients was 469 days with a range of 40 to 1596 days as measured from the end of radiation.

**Table 1:** Patient Characteristics

Characteristic	Total	KUMC	Thomas Jefferson
Total	212	92	120

Gender			

Male	124	69	55

Female	88	23	65

Age (median, years)	58	58	56

Tumor Site			

Oral cavity	29	17	12

Oropharynx	89	39	50

Larynx	54	29	25

Hypopharynx	3	2	1

Salivary Gland	13	2	11

Nasal-Sinus	9	1	8

Unknown primary	5	1	4

Stage			

T0	13	6	7

T1-2	66	28	38

T3-4	120	58	62

Recurrent	13	0	13

N0	78	28	50

N1	36	18	18

N2-3	98	46	52

Chemotherapy	159	59	100

Surgical resection	42	18	24

SUVs were available for 152 of the masses and 79 of the lymph nodes, 52 patients did not have a reported SUV (results for these patients were reported as positive or negative). The median maximum SUV was 8.75 with a range of 1 to 41 for the main tumor mass. The median maximum SUV was 5.95 with a range of 2 to 25 for the lymph node, when multiple lymph nodes were found, only the lymph node with the highest SUV was used.

**Figure 1:**
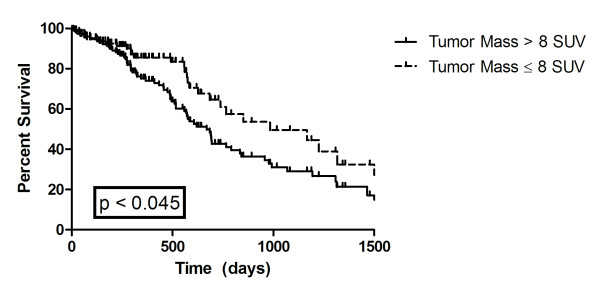
**Kaplan Meier Overall Survival**. Figure 1 shows overall survival by mass SUV greater and lesser than 8. Curves are significantly different (p < 0.045).

At last follow-up, 119 patients were still alive; Kaplan-Meier median overall survival for the entire cohort was 886 days, Kaplan-Meier median disease free survival for entire cohort was 726 days. When SUV of the tumor mass was analyzed in terms of overall survival using log-rank (Mantel-Cox) test, it was found that SUV > 8 was statistically significant for a worse overall survival (p < 0.045) with an overall survival of 669 days for patients with an SUV of the mass greater than 8 and a median overall survival of 984 days for patients with a SUV of the mass less than 8. Figure 1.

**Figure 2:**
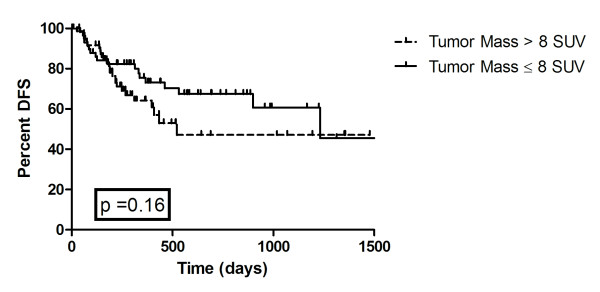
**Kaplan Meier Disease Free Survival**. Figure 2 represents disease free survival by mass SUV greater and lesser than 8. The curves are not statistically significantly different (p = 0.16).

**Figure 3:**
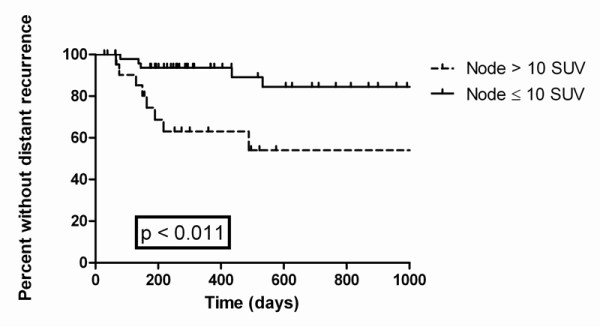
**Kaplan Meier Time to Distant Recurrence**. This graph shows time to distant recurrence with nodal SUV greater to and lesser than 10. The curves are statistically significantly different (p < 0.011).

Seventy-eight patients had a recurrence, 35 distant and 43 local, with a median time to recurrence of 135 days. One hundred and thirty one (61.7%) had local control with no evidence of disease at last follow-up or at time of death from non-cancer causes. Twenty patients had persistent disease after radiation, 5 of these patients had distant recurrence as well. There was a trend for patients with an SUV of the mass ≤8 to have improved local control, but this did not reach statistical significance (p = 0.16) Figure 2. The maximum lymph node SUV was found to be predictive for distant failure (p < 0.0001), but not for overall or local failure. Median lymph node SUV was 10.0 for patients with distant failure vs. 5.2 without. Figure 3.

One hundred and ten patients had a neck dissection performed after the PET scan prior to radiotherapy. PET scan in these patients was obtained a median of 12 days prior to the surgical neck dissection (range 3 to 91 days). For these 110 patients, the radiographic lymph node status was compared to the final surgical pathology. Four false positives and 5 false negatives were found giving a sensitivity of 92.5%, specificity of 90.7%, positive predictive value (PPV) of 93.9% and a negative predictive value (NPV) of 88.6%. Of the 5 false negative PET scans, there was 1 T1 base of tongue cancer, 1 T2 parotid, 1 T2 oral tongue, and 2 T4 oral cavity. All patients with a false negative PET scan had one lymph node positive for metastatic disease found on subsequent neck dissection. For the false positive results, there was 1 T2 oral tongue, 1 T4 oral cavity, 1 T4 larynx, and 1 T2 tonsil. Neck dissections for the 4 false positive PET scans showed no positive lymph nodes with a median of 23 (range 9 to 27) nodes dissected.

Twenty-two patients had ECE of a lymph node found on neck dissection; fifteen of these patients had reported SUV of the lymph node. The median SUV of these lymph nodes was 11.9 versus 5.0 for lymph nodes without ECE, which was significantly different (p < 0.0007) on a student's T-test. ECE was found in 6 patients with N1 nodal disease and 16 patients with N2-3 nodal disease. On a repeated measures ANOVA, there was no difference in median SUV of the lymph node based on nodal status (N1 versus N2-3).

Imaging modality was a PET scan in 119 patients and a PET/CT scan in 93 patients. For the 110 patients with post-PET neck dissections, 55 had PET-CT scans and 55 had PET scans. When analyzed for any differences between the imaging modalities by nodal staging, PET/CT scans had 2 false positive results and 0 false negatives while PET without CT fusion had 2 false positives and 5 false negatives. The sensitivity for the PET/CT was 100% with a specificity of 90.1% and a PPV of 94% and NPV of 100%. For PET alone, the sensitivity was 85.7%, specificity 90%, PPV of 93.4% and NPV of 78.2%. Using a student's T-test, PET and PET/CT were not statistically significantly different in overall ability to determine lymph node status (p = 0.089).

## Discussion

Several recent innovations have been introduced in efforts to improve the clinical outcome in the treatment of HNC. New radiotherapy technologies such as IMRT and IGRT have improved radiotherapy delivery and new surgical techniques including sentinel lymph node biopsy also have potential for improving HNC therapy. In addition, increased implementation of diagnostic exams such as PET and PET/CT scans have the potential to improve treatment outcomes by providing improved lymph node staging, prognostic, and perhaps predictive factors.

We found excellent positive and negative predictive value for PET scans in determining lymph node status. This finding is in line with several other reports [5,9-17]. Table 2. These findings have potential clinical implication in that it may be possible to use staging PET scan results in stratifying patients who should go on to receive neck dissections. Ng et al. [11] found false negative PET scan results depended on the T stage of the tumor with false negative PET scans in 6.7, 10.8, 13.3, and 25% for T1-4 tumors respectively. Our results also demonstrate the low false negative rate in staging PET scans, although we did not find a relation between T stage and false negative rates, likely secondary to the low number of false negative PET scans. A recent meta-anaylsis [28] examining the results of PET scan in cervical lymph node staging found lower sensitivity (79%) and specificity (86%) results than our study. Some explanations for this include the fact that most of the studies in the meta-analysis were older and used PET scan alone rather than combined PET/CT.

**Table 2:** Results for Staging with PET

Study	Patients	Sensitivity	Specificity
Murakami (8)	23	79	99

Ng (10)	134	47	92

Fleming (17)	67	86	94

Hannah (11)	48	82	100

Schwartz (15)	20	100	96

Zanation (4)	97	93	89

Roh (21)	167	87	93

Kyzas (28)	1236	79	86

Kubicek*	110	93	91

* Current Study

Several authors have examined if PET/CT is superior to PET alone in nodal staging [29-31]. Bransetter et al. [30] reported that PET/CT was superior to PET alone in HNC lesions, and Schoder et al. [32] arrived at the same conclusion. The overall accuracy in determining lymph node status was not statistically significantly better for PET/CT in this study, but there was a trend towards significance. Although PET/CT had several false-positive results there were no false negatives. The added accuracy of CT fusion with the PET scan, especially in terms of reducing the number of false negative scans, is important in the use of PET/CT to limit surgical neck dissection.

This report and others can help determine the best use for PET scans in the nodal staging of HNC. Our report lends strength to the previously published studies regarding the excellent predictive value of PET scans in determining lymph node status. Having adequate knowledge of lymph node status without the aid of surgical dissection has several potential areas of clinical benefit. Neck dissections add significant morbidity in HNC patients [33] and PET scans may be able to determine which patients do not require a full dissection. If further evidence, perhaps in the form of a prospective trial, can confirm the accuracy of PET scans in lymph node staging, it would be possible to use imaging in lieu of surgery for determining lymph node status.

The role of maximum SUV as a prognostic factor is controversial. Several reports have found a particular SUV value associated with a worse outcome [18-24]. However, other authors did not report this association [26,27]. One factor in the mixed findings of maximum SUV and outcome may be related to the relatively small number of patients in many of these studies. Our data found that SUV of the tumor mass > 8 was associated with worse overall survival. Interestingly, we did not find an association between tumor mass SUV and local control, likely secondary to several late relapses. Fewer reports have focused on the prognostic information of nodal SUV. Schwartz et al. [19] did not find any association between nodal SUV and outcome in 36 patients, Brun et al [20] also failed to find any association and also had 36 patients. Liao et al. [27] examined only oral cavity patients with positive lymph nodes and found that a lymph node SUV of 5.4 predicted for worse outcomes. We found an association between maximum SUV of the lymph node and outcome with nodal SUV > 10 more likely to have distant failure in 79 patients with nodal SUV. One explanation of the discrepancy between our data and that of Schwartz et al. and Brun et al. could be the larger number of patients which would give our study power to detect a true finding.

Although prognostic information, such as tumor mass SUV, is useful, it is not used for management changes. However, the association between maximum lymph node SUV and distant failure has possible clinical implications. One of the current trends (although still unproven) in HNC is neoadjuvant chemotherapy with TPF [34,35]. If further reports confirm the finding that maximum lymph node SUV > 10 has a higher likelihood of distant failure but not a higher likelihood of local failure, this would be a possible stratification factor for which patients should receive neoadjuvant TPF rather than concurrent therapy.

We also found that maximum SUV was predictive of ECE. ECE is an established risk factor and is one of the indications (the other being positive margins) in determining which patients post-operatively benefit from combined chemoradiotherapy rather than radiotherapy alone [36]. For patients receiving definitive radiotherapy, it may make be beneficial to treat higher SUV lymph nodes (which are more likely to harbor ECE) to a higher dose.

The results of this study need to be interpreted with caution; it is retrospective in nature and includes a variety of head and neck cancer subtypes. In this regard, our study has the same flaws as the majority of other reports examining the utility of FDG-PET in the staging of head and neck cancer. However, this study does have some improved clinical utility secondary to its size (212 PET scans analyzed) and the multi-institution nature of this report. We acknowledge that most of the information gathered are hypotheses generating and that further studies, perhaps in a prospective randomized fashion, will be required to truly determine the utility for PET scans in HNC. Until such studies are completed, large retrospective such as this are needed to provide the best information at this time possible.

## Conclusions

FDG-PET scanning has good accuracy and predictive value in determining lymph node status. SUV of the tumor mass is prognostic for overall survival. SUV of the lymph node is prognostic for ECE and also for distant recurrence. Patients with higher lymph node SUVs treated with definitive radiation may warrant higher radiotherapy doses to overcome a greater likelihood of ECE. Nodal SUV may be used to predict patients who would be more likely to benefit from induction chemotherapy.

## Competing interests

The authors declare that they have no competing interests. There was no funding for this research project.

## Authors' contributions

GK: wrote manuscript, data analysis, CC: data retrieval and chart review, SF: edited manuscript, assistance with data analysis, FW: edited manuscript, ER: edited manuscript, CI: edited manuscript, RD: edited manuscript, MM: edited manuscript, project design. All authors read and approved the final manuscript
